# The CD105:CD106 microparticle ratio is CD106 dominant in polycystic ovary syndrome compared to type 2 diabetes and healthy subjects

**DOI:** 10.1007/s12020-019-02059-9

**Published:** 2019-08-27

**Authors:** Ahmed Al-Qaissi, Saeed Alqarni, Zeeshan Javed, Stephen L. Atkin, Thozhukat Sathyapalan, Rebecca V. Vince, Leigh A. Madden

**Affiliations:** 1 Department of Academic Diabetes, Endocrinology and Metabolism, Hull York Medical School, The University of Hull, Hull, UK; 2 Department of Biomedical Science, The University of Hull, Hull, UK; 3 Royal College Surgeons Ireland, Manama, Bahrain; 4 Department of Sport, Health and Exercise Science, The University of Hull, Hull, UK

**Keywords:** Polycystic ovary syndrome, Endothelial microparticles, VCAM-1

## Abstract

**Background:**

A retrospective analysis was carried out from patients and controls during the past 5 years from a series of studies investigating endothelial microparticles (MP).

**Methods:**

In total, 319 samples from 207 individuals were included in this analysis, from patients with type 2 diabetes (T2D, *n* = 105), women with polycystic ovary syndrome (PCOS, *n* = 145) and healthy volunteers (*n* = 69). All data were generated via the same flow cytometry protocol with the same antibody clones. Endothelial markers CD105 (Endoglin) and CD106 (Vascular cell adhesion molecule-1) were used to enumerate MP in venous blood.

**Results:**

The ratio of CD105MP:CD106MP was significantly different between groups (*F* = 63.43, *p* < 0.0001). Women with PCOS were found to have a median CD105MP:CD106MP ratio of 0.40 (IQR 0.24–0.57), suggesting approximately two CD106MP were found per CD105MP. The T2D group showed a median ratio of 2.32 (1.51–3.69) whereas in healthy volunteers the ratio was 2.21 (1.63–3.55). Serum intercellular adhesion molecule-1 was also shown to be significantly increased in PCOS when compared with control or T2D groups (*F* = 14.5, *p* < 0.001).

**Conclusion:**

These data suggest that women with PCOS have an altered endothelial MP release in favour of CD106. Thus a potential activated endothelial state exists in women with PCOS with a shift towards a predominantly CD106MP profile.

## Introduction

Polycystic ovary syndrome (PCOS) is the most common endocrine disorder amongst women of reproductive age that commonly presents with menstrual irregularities, ovulatory dysfunction and clinical and/or biochemical hyperandrogenism [1, 2]. PCOS shares several risk factors with metabolic syndrome such as insulin resistance, impaired glucose tolerance, obesity, hypertension and dyslipidaemia; thus these patients are at high risk of developing diabetes mellitus and cardiovascular disease [3]. In addition, PCOS is associated with endothelial dysfunction that is one of the earliest and most prominent signs and a prognostic marker of future atheromatous cardiovascular disease [4]. Dysfunctional endothelium results in abnormal activation and adhesion of platelets and leucocytes, as well as the release of cytokines, thus increasing permeability of the vessel wall to oxidised lipoproteins and inflammatory mediators, resulting in arterial wall structural damage and atherosclerotic plaque formation [5, 6]. Multiple metabolic cardiovascular risk factors, common in PCOS, accelerate the process of endothelial dysfunction portending an increased risk of cardiovascular events in these patients [7].

Cell derived microparticles (MP) are a heterogeneous population of extracellular vesicles (0.1–1 μm) that are released from the cell membrane during cell activation and apoptosis and contribute to the induction of endothelial cell modifications, differentiation, inflammation and angiogenesis [8]. MPs are also vital messengers in inter-cellular communications [8]. They have been suggested to play a significant role in endothelial dysfunction, cellular inflammation, coagulation and angiogenesis, and thus predispose to cardiovascular diseases by perturbing vascular homoeostasis. Recent trials have revealed that PCOS patients have higher levels of endothelial microparticles (EMP) [8]. EMP levels are also increased in a variety of cardiovascular and atherothrombotic diseases such as diabetes, obesity, end-stage renal disease, acute coronary syndromes, cancers, inflammatory disorders and autoimmune diseases [9]. In both acute coronary syndrome and diabetic patients, EMP correlate positively with the extent and severity of stenosis and represent a more robust predictor of the occurrence of cardiovascular events in diabetic patients compared with traditional markers of endothelial activation [10].

The physiological relevance of MP is becoming clearer; however, there are variations across laboratories in terms of sample handling and analysis that have been addressed to some extent by protocol standardisation [11] although a large variation in reported quantified values exists. Most studies, if not all, report on either a single specific marker or multiple (usually dual) labelling to allow differentiation between cells of origin, and often include Annexin V as an apoptotic marker.

The aim of the study was to investigate EMP populations as ratios of constitutively expressed (CD105; Endoglin) versus activation markers (CD106; Vascular cell adhesion molecule-1). This new method of analysis removes the variations mentioned above by standardising measurements. Data included in the study was produced from a range of previous studies on women with PCOS, patients with type 2 Diabetes (T2D) and healthy controls.

## Methods

### Patient cohorts

Patients were recruited into studies between 2014 and 2019, where EMP were determined prior to any interventions. Four studies analysed here consisted of women with PCOS (PCOS groups 1–4). Two further studies recruited patients with T2D (T2D groups 1 and 2) and control groups (control groups 1 and 2). Informed consent was given in accordance with favourable ethical opinion for each separate study (REC references; 14/YH/1125, 17/YH/0118, 16/NW/0518, 16/YH/0183). All volunteers were treated in accordance of the principles of the Declaration of Helsinki.

### EMP analysis

Venous blood samples were drawn from the antecubital vein into citrated sample tubes (Vacutainer, Greiner, UK) and analysed within 2 h from the time of collection. Platelet free plasma was prepared via a double centrifugation step, first the blood sample tubes were centrifuged at 400 × *g* for 10 min. Platelet rich plasma was removed from the tube and centrifuged again at 10,000 × *g* for 10 min to remove platelets. The platelet free plasma (25 μL) was incubated with 5 μL of either an anti-human CD105:FITC (clone SN6, Bio-Rad, UK) or an anti-human CD106:FITC (clone 1.G11B1, Bio-Rad, UK) for 30 min in the dark at room temperature. Counting beads (25 μL, Accucheck counting beads, Invitrogen, UK) and 0.2 μm filtered, sterile phosphate buffered saline (150 μL) were then added prior to flow cytometry (BD FACSCalibur, CELLQuest software). A MP gate was defined according to side scatter using Megamix SSc beads (Biocytex, France) following the International Society for Thrombosis and Haemostasis working group protocol [11] using a validated flow cytometer for MP analysis.

### Soluble intercellular adhesion molecule-1 (ICAM-1) analysis

Blood samples were collected by standard venepuncture from an antecubital vein into serum collection tubes (Greiner, UK). Samples were allowed to clot (30 min) then centrifuged for 10 min at 2000 × *g*. Serum was carefully removed and stored at −80 °C until analysis. Soluble ICAM-1 was quantified within these samples using a commercially available kit (BMS-241, Thermo-Fisher, UK) following the manufacturers’ instructions.

### Statistical analysis

Groups were merged for statistical analysis into PCOS, T2D and non-diabetic controls. Analysis of variance (one-way ANOVA) was used for comparison of CD105:CD106MP ratios across groups and between males and females in the mixed sex groups and BMI with significance set at 0.05. Post hoc Tukey's test was carried out to identify differences between the groups. Pearson’s correlation was used to investigate the relationship of the EMP ratio with age and BMI.

## Results

### Demographic data

The demographic data from the individual study groups are shown in Table 1. Comparisons were made between age, BMI and male/female recruits. Groups were merged for subsequent analyses.

**Table 1:** Demographic data

Group	PCOS 1	PCOS 2	PCOS 3	PCOS 4	T2D 1	T2D 2	T2D Control 1	T2D Control 2
Age	31.0 ± 5.8	26.5 ± 5.0	28.6 ± 5.5	29.1 ± 7.5	61.4 ± 10.8	62.0 ± 7.0	56.3 ± 9.8	55.0 ± 10
BMI	38.8 ± 7.8	37.2 ± 6.1	35.4 ± 10	33.8 ± 7.2	26.8 ± 5.4	32 ± 4	31.6 ± 5.8	28 ± 3
% Male	0	0	0	0	70.9	52.2	45.0	45.5

### CD105MP:CD106MP ratio

**Fig. 1:**
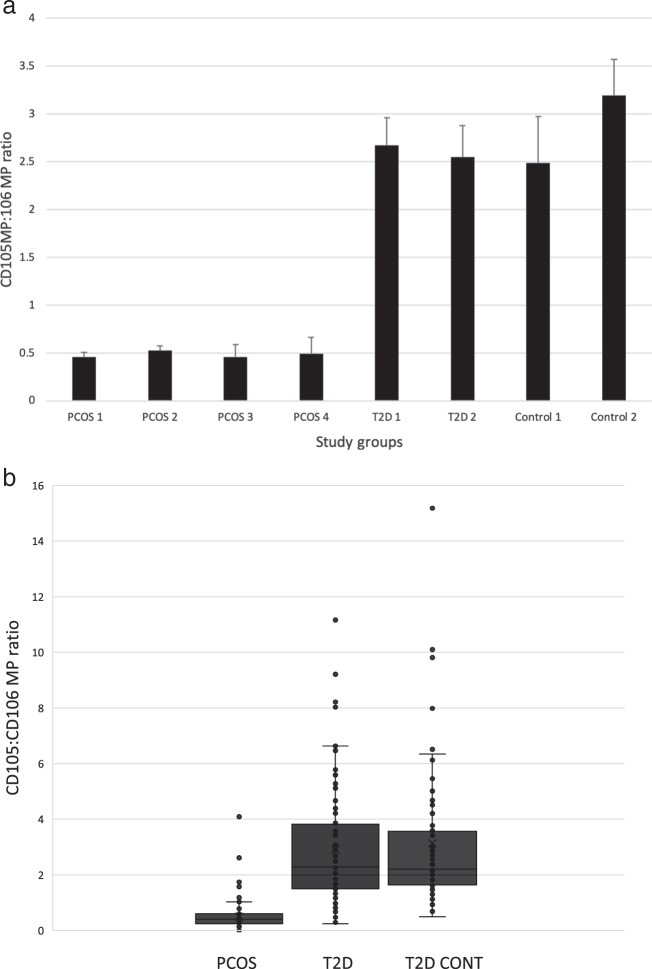
**a** The CD105:CD106 MP ratio across individual studies. Groups were PCOS 1 groups 1–4, T2D groups 1 and 2 and control groups 1 and 2. Error bars represent SEM. **b** Box plot showing combined data for PCOS, T2D and controls groups. Horizontal bar within the box represents the median, ‘x’ denotes mean

The CD105MP:CD106MP ratio for the individual groups is shown in Fig. 1a and merged group data in Fig. 1b. Between groups analysis (PCOS, T2D and controls) showed a highly significant difference between the groups (*F* = 63.43, *p* < 0.0001). Post-hoc analysis showed in women with PCOS the CD105MP:CD106MP ratio was significantly different to both the T2D groups (*p* < 0.0001) and the control groups (*p* < 0.0001). No difference was found between T2D groups and control groups (*p* = 0.59).

### Male/female ratio in T2D and control groups

No difference was observed in the ratio of CD105MP:CD106MP between male and female subjects combined from the T2D and control populations (*F* = 0.015, *p* = 0.9).

### BMI

There was an overall significant difference in BMI between the groups (*F* = 32.64, *p* < 0.001). Combined data showed a median (IQR) of 26.6 (24.7–30.2) in the T2D control group, 31.4 (28–34.6) in the T2D group and 36.1 (30.48–41.5) in the PCOS group. Within groups there was a weak to moderate correlation between CD105:CD106 ratio and BMI in the control group (*r* = 0.40) and no correlation in either the T2D group (*r* = 0.07) or the PCOS group (*r* = 0.03).

### Age

T2D group 1/2 and control groups 1/2 were age matched but significantly older than PCOS groups (*p* < 0.001). Within groups there was no correlation between age and CD105:CD106MP ratio (*r* = 0.17 [controls], *r* = 0.13 [T2D] and *r* = 0.07 [PCOS]).

### Soluble ICAM-1

**Fig. 2:**
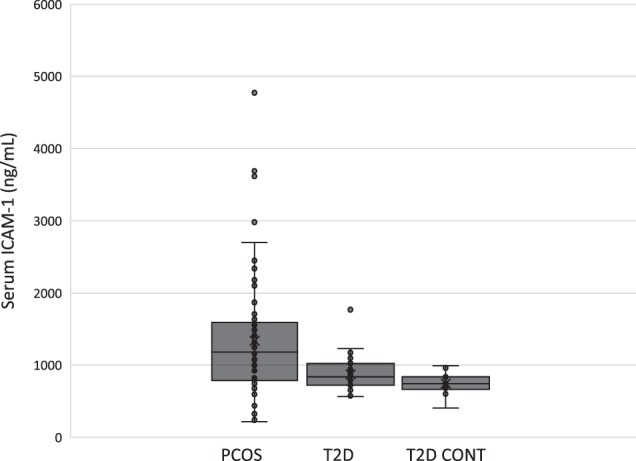
Serum ICAM-1 levels in PCOS (*n* = 37), T2D (*n* = 56) and control groups (*n* = 18) as determined by ELISA. Horizontal bar within the box represents the median, ‘x’ denotes mean

Serum levels of ICAM-1 were quantified by ELISA on a number of samples across PCOS (*n* = 37), T2D (*n* = 56) and control groups (*n* = 18). PCOS was associated with significantly higher serum ICAM-1 (median 1318 ng/ml [IQR 792–1581]) than either T2D (841 ng/ml [726–1010]) or control (746 ng/ml [668–823]) groups (*F* = 14.5, *p* < 0.001) as shown in Fig. 2. There was no significant difference observed between T2D and control groups.

### Markers of insulin resistance

Where data were available there was no correlation between HOMA-IR and CD105:CD106MP within the PCOS group (*r* = 0.004) nor was there any observed correlation between HbA1c levels and CD105:CD106MP ratio within the T2D group (*r* = 0.132) or the control group (*r* = 0.334). Also within a PCOS group there was no correlation between triglycerides and CD105:CD106MP ratio (*r* = 0.219).

## Discussion

Endothelial MP may provide an insight into the state of the endothelium in vivo. The use of MP ratios as presented here offers a novel analysis method to compare data generated across studies. The analysis of MP ratios herein could aid in the distinction between disease and healthy states. Previous studies have focussed on platelet MP in relation to PCOS and found elevated levels [12–14]. Endothelial, platelet and leucocyte-derived MP were also shown to be significantly higher in women with PCOS than controls, suggesting MP could be a biomarker of PCOS [8]. In the present study this concept is expanded upon to show that ratios of EMP can be used to discriminate between conditions associated with endothelial dysfunction, confirming the notion of EMP as biomarkers in PCOS. Based on the analysis presented, it is suggested that utilising a particular EMP ratio, for example CD105:CD106 would allow for comparisons to be made across multiple sites and studies where it has been recently shown that even when using a standardised technique and reagents individual sample variability can be as high as 37% [15]. Other markers which may be useful could include other activation markers such as CD54 or CD62E. Serum levels of ICAM-1 were shown here to be significantly higher than either T2D or control groups and women with PCOS have previously been shown to have increased serum levels of inflammatory markers such as CD62E and VCAM-1 in addition to ICAM-1 [16]. Furthermore, levels of pro-inflammatory cytokines, especially interleukin-18, which stimulates the synthesis of interleukin-6 are increased [17].

The data presented here suggest a shift in EMP release in women with PCOS towards a CD106 dominant profile, whereas all other groups showed a dominant CD105 profile. Women with PCOS had on average an approximate 2:1 ratio of CD106MP to CD105MP. This may be indicative of endothelial cell activation or endothelial dysfunction over and above anything observed within the diabetes groups or a reflection of a differing EMP release mechanism in these women. Obviously PCOS only occurs in females and so the observed data could suggest a hormonal or medication dependent change in EMP release however the T2D and non-diabetic control groups analysed included females and analysis showed no difference in CD105:CD106MP ratio within the two diabetes with controls studies when accounting for sex alone.

Though gender did not appear to influence EMP ratio, age and BMI might be anticipated to be influencing factors as endothelial function becomes poorer with age [18]. However, we found no significant difference in CD105:CD106 MP ratio between patients with T2D and healthy age-matched controls, suggesting that the difference seen in women here were due to the prevalence of PCOS in those groups. Furthermore, there were no significant within group correlations between age or BMI and CD105:CD106MP ratio.

The T2D CD105:CD106 MP ratio did not differ significantly from the control group despite T2D being associated with impaired endothelial function. The T2D groups were on medication for diabetes but the PCOS groups were not on any form of insulin control medication. The observed similarity in ratio of the T2D and control groups could therefore be a consequence of medication and would be a limitation of the study warranting further investigation. Serum ICAM-1 was determined in a subset of patients and showed that women with PCOS had a significantly higher serum ICAM-1 concentration thus confirming altered endothelial cell function or activation.

PCOS is associated with chronic low grade inflammation that may contribute to an increased risk of future cardiovascular events, and may contribute to the underlying pathology of the endocrine and metabolic abnormalities including endothelial dysfunction that is found [19]. It has also been demonstrated in several studies that PCOS is directly correlated with endothelial dysfunction and vascular anomalies and patients are at increased cardiovascular risk [16, 20–22]. Paradisi et al. [23] reported that women with PCOS had a significant (ca. 50%) reduction in endothelium-mediated vasodilation and in addition insulin resistance and elevated testosterone levels were associated with this reduction. Moreover, a study on PCOS women that used cardiovascular magnetic resonance to measure endothelial function of vessels established that endothelial dysfunction was significantly decreased (*p* < 0.01) with flow-mediated dilatation [24].

Endothelial dysfunction can also be evaluated by quantifying specific indicators of coagulation such as plasminogen activator inhibitor-1 (PAI-1), markers of inflammation (e.g. ICAM-1 and VCAM-1, or circulating markers derived from the endothelium such as ADMA (asymmetric dimethylarginine). The levels of such markers of endothelial dysfunction (PAI-1, ADMA, VCAM-1, and ICAM-1) have also shown to be higher in PCOS patients as compared with controls [25]. In a meta-analysis PCOS patients were reported to have a significantly higher prevalence of endothelial dysfunction (as measured by low flow mediated dilatation) with subsequent higher risk of future cardiovascular events as compared with their age and weight matched controls, effects that were independent of their age and normal BMI [26]. A recent study showed that platelet-associated arginase activity was significantly higher in PCOS compared with a control group and that platelet MP were increased in PCOS and were the main source of circulating arginase activity [27]. This could impact vascular homoeostasis and lead to cardiovascular disease via arginase competition for l-arginine with endothelial nitric oxide synthase [27, 28].

## Conclusion

This report highlights the use of endothelial cell derived MP ratios as a way to compare and contrast multiple studies of endothelial function and demonstrates that women with PCOS have an altered MP profile with a CD106 dominant phenotype.
